# Protective Effects of Evogliptin on Steatohepatitis in High-Fat-Fed Mice

**DOI:** 10.3390/ijms21186743

**Published:** 2020-09-14

**Authors:** Jin Hyun Kim, Si Jung Jang, Gu Seob Roh, Hyun Seop Cho, Heeyoung Kang, Soo Kyoung Kim

**Affiliations:** 1 Biomedical Research Institute, Gyeongsang National University Hospital, Jinju 52727, Korea; ajini7044@hanmail.net (J.H.K.); sjjang@gnu.ac.kr (S.J.J.); 2 Institute of Health Sciences, Gyeongsang National University, Jinju 52727, Korea; anaroh@gnu.ac.kr (G.S.R.); mars@hanmail.net (H.S.C.); miranda75@naver.com (H.K.); 3 Department of Anatomy and Neurobiology, College of Medicine, Gyeongsang National University, Jinju 52727, Korea; 4 Department of Internal Medicine, Gyeongsang National University Hospital, Jinju 52727, Korea; 5 Department of Neurology, Gyeongsang National University Hospital, Jinju 52727, Korea; 6 College of Medicine, Gyeongsang National University, Jinju 52727, Korea

**Keywords:** steatohepatitis, obesity, evogliptin, autophagy, mitophagy

## Abstract

There are few studies on the effects of dipeptidyl peptidase-4 inhibitors on steatohepatitis. We explored whether evogliptin (Evo), a dipeptidyl peptidase-4 inhibitor, protects against steatohepatitis in a high-fat diet (HFD)-fed mice and whether these effects involve modulation of mitophagy. Adult male C57BL/J mice were divided into the normal diet (ND), HFD (45% of energy from fat) with Evo (250 mg/kg) (HFD + Evo), and HFD groups at 4 weeks of age and were sacrificed at 20 weeks of age. The HFD group showed hepatic lipid accumulation; this was decreased in the Evo + HFD group. There was an increased 8-hydroxydeoxyguanosine (8-OHDG) expression in the HFD group compared to ND mice. However, 8-OHDG expression levels were significantly decreased in the HFD + Evo group. Expressions of the mitophagy markers PTEN-induced kinase 1 (PINK1), Parkin, and BNIP-3 (BCL2 Interacting Protein 3) were significantly increased in the HFD group. However, the expressions of these markers were lower in the HFD + Evo group than that in the HFD group. Phospho-Akt was upregulated and p53 was downregulated in the HFD + Evo group compared to the HFD group. Evogliptin may alleviate steatohepatitis in HFD-fed mice by ameliorating steatosis and oxidative stress and by modulating mitophagy in the liver.

## 1. Introduction

Nonalcoholic fatty liver disease (NAFLD) ranges from simple steatosis to nonalcoholic steatohepatitis and/or cirrhosis [1]. Development of NAFLD is related to insulin resistance, oxidative stress, inflammation, mitochondrial dysfunction, and fibrosis [1,2]. Especially, mitochondrial function increases with fat overload of hepatocyte. However, sustained fat overload and deposition lead to impairment of mitochondrial function [3].

Autophagy removes unnecessary or dysfunctional cellular components by delivering them to lysosomes for degradation [4]. Thus, autophagy is crucial for maintaining cell function and is required for a functional liver. Autophagy is typically nonselective but can be selective under certain conditions [4]. Mitophagy, the selective degradation of damaged mitochondria, is one type of selective autophagy. Phosphatase and tensin homolog-induced kinase 1 (PINK1)/Parkin (E3 ubiquitin ligase)-mediated mitophagy promotes selective degradation of damaged mitochondria [5,6]. Accumulation of PINK1 in the outer membrane of damaged mitochondria recruits cytoplasmic Parkin to those mitochondria, resulting in LC3-mediated fragmentation and engulfment of mitochondria by autophagosomes [5,6]. Previous studies reported that mitophagy alleviates liver steatosis and that impairment of mitophagy deteriorates the NAFLD [7,8]. However, Pang et al. reported that mitophagy promotes apoptosis of hepatocytes in vitro while its inhibition suppresses their apoptosis [9]. Therefore, the role of mitophagy in NAFLD is ambiguous, being both protective and deleterious.

Dipeptidyl peptidase-4 (DPP-4) inhibitors have been approved for the treatment of type 2 diabetes. Previous studies showed that inhibition of DPP-4 prevents hepatic steatosis in animal models [10,11,12,13]. Evogliptin (Evo), a novel DPP-4 inhibitor, was recently approved for type 2 diabetes by the South Korean Ministry of Food and Drug Safety. It has been found to prevent hepatic steatosis in animal models by suppression of de novo lipogenesis [14]. However, the precise underlying mechanisms are unclear. Thus, we investigated the effects of Evo on steatohepatitis in mice with a high-fat diet (HFD) and the roles of autophagy and mitophagy in any such effect.

## 2. Results

### 2.1. Effect of Evogliptin on Body Weight, Epididymal Fat Pad Weight, Glucose Level, Food Intake, Serum Insulin, and Liver Enzyme in HFD Mice

**Figure 1:**
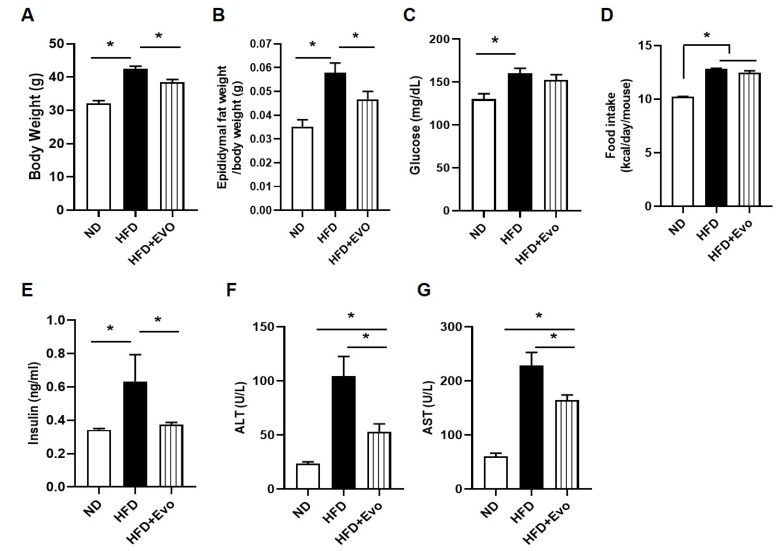
Effect of evogliptin on body and fat weights in mice with HFD: mice were euthanized 16 weeks after HFD feeding. (**A**) Body weight, (**B**) epididymal fat weight, (**C**) glucose level, (**D**) food intake, and serum (**E**) insulin, (**F**) ALT, and (**G**) AST levels at 16 weeks after HFD feeding. Values are mean ± SEM. * *p* < 0.05, HFD: high-fat diet; ND: normal diet. ALT: Alanine aminotransferase; AST: Aspartate aminotransferase.

There were no differences in body weight among the three groups at baseline. The body weight of the HFD group was significantly increased compared to the ND group, and the HFD + Evo group had a significantly lower body weight than the HFD group and similar body weight to that of the ND group at 16 weeks after HFD feeding (Figure 1A). The epididymal fat weight was significantly increased in the HFD group compared to the ND group and was decreased in the HFD + Evo group to a similar level to the ND group (Figure 1B). There were no significant differences in the glucose level at baseline among the three groups. At 16 weeks after HFD feeding, the glucose level significantly increased in the HFD group compared with the ND group. The level in the HFD + Evo group was lower than in the HFD + Evo group but was not significant (Figure 1C). Overall, the HFD and HFD + Evo groups consumed more energy per day than the ND group, whereas energy consumption did not show a significant difference between the HFD and HFD + Evo groups (Figure 1D). The insulin level was significantly increased in the HFD group, and evogliptin improved hyperinsulinemia (Figure 1E). The levels of serum aspartate aminotransferase (AST) and alanine aminotransferse (ALT) were significantly increased in the HFD group and significantly reduced in the HFD + Evo group (Figure 1E,F).

### 2.2. Effect of Evogliptin on Hepatic Steatohepatitis in HFD Mice

**Figure 2:**
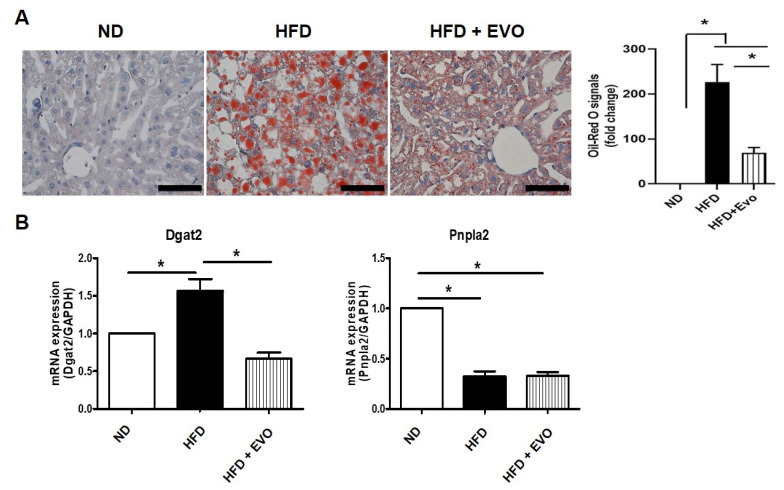
Evogliptin decreased hepatic lipid accumulation: (**A**) liver sections stained with Oil Red O. Scale bar, 50 μm. (**B**) mRNA levels of lipid metabolism-related genes as determined by qPCR: GAPDH was used as an internal control. Values are mean ± SEM. * *p* < 0.05, HFD: high-fat diet; ND: normal diet.

In the ND group, the liver morphology was normal and there was no Oil Red O-positive staining. However, in the HFD group, Oil Red O-positive lipid droplets were distributed throughout the liver and pathological features of steatohepatitis including steatosis and lipogranulomas were evident. The HFD + Evo group showed decreased hepatic lipid accumulation and injured liver parenchyma compared to those in the HFD group (Figure 2A). The expression of Dgat2, which catalyzes the final step in triacylglycerol (TG) synthesis, was significantly increased in the HFD group compared to the ND group and was decreased in the HFD + Evo group. (Figure 2B). The expression of Patatin-like phospholipase domain-containing 2 (Pnpla2), which catalyzes intracellular hydrolysis of stored triglycerides, was significantly downregulated in the HFD and HFD + Evo groups compared to the ND group (Figure 2B). Therefore, these results suggest that Evo ameliorates HFD-induced steatohepatitis by modulating the expression of lipid metabolism-associated genes.

### 2.3. Effect of Evogliptin on Hepatic Morphological Changes and Fibrosis by HFD

**Figure 3:**
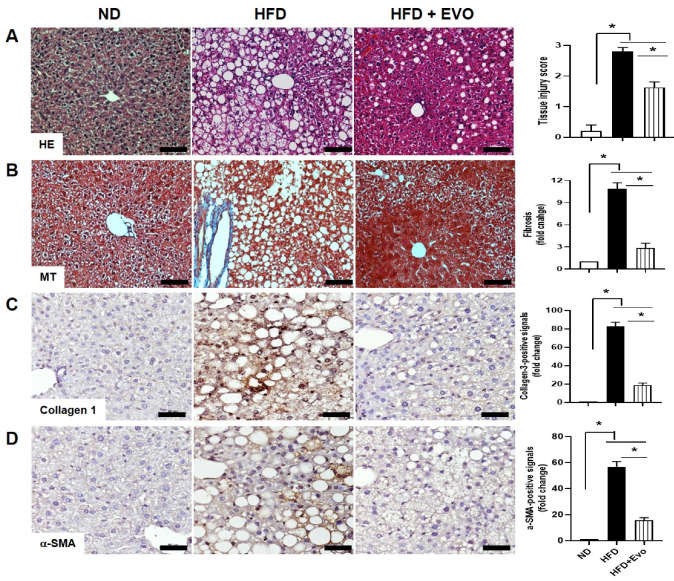
HFD-induced hepatic steatosis and fibrosis were attenuated by evogliptin. Representative liver images from each group by H&E (**A**), MT staining (**B**), collagen-3 (**C**), and α-SMA (**D**). Scale bar, 100 μm for H&E and MT staining. Scale bar, 50 μm for collagen-III and α-SMA staining. Data are means ± SD. * *p* < 0.05 compared to the indicated groups. HFD: high-fat diet; H&E: haematoxylin and eosin; MT: Masson’s trichrome; α-SMA: α- Smooth Muscle antibody, ND: normal diet.

Histological analyses by haematoxylin and eosin (HE) staining demonstrated that hepatic steatosis (steatosis and hepatocellular ballooning) was decreased in the HFD group treated with Evo (Figure 3A). Accumulation of vascular collagen fibers by Masson’s trichrome (MT) staining was observed in the HFD group, but Evo improved HFD-induced vascular fibrosis in the liver (Figure 3B). To confirm the effect of Evo on HFD-induced hepatic fibrosis, we performed to staining of α- Smooth Muscle antibody (SMA) and collagen III. Both α-SMA and collagen III-positive signals were increased in the HFD group compared to ND and were significantly decreased in the HFD + Evo group (Figure 3C,D).

### 2.4. Effects of Evogliptin on Hepatic Autophagy in HFD Mice

**Figure 4:**
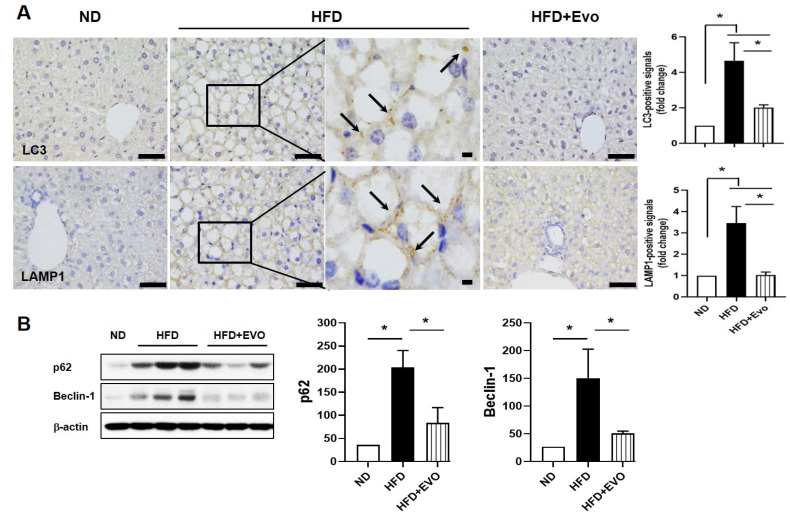
Evogliptin decreased the levels of autophagy-related proteins. (**A**) Representative immunohistochemical liver images for LC3 and LAMP-1 from each group: HFD-induced positive signals (black box) were magnified and indicated as arrows. Scale bar, 50 μm. (**B**) Protein expressions related to autophagy and the quantitative results: values are mean ± SEM. * *p* < 0.05, HFD: high-fat diet; ND: normal diet.

Autophagy is associated with increased TG levels and inhibition of β-oxidation [15]. Therefore, we examined the effects of Evo on autophagy in HFD mice. LC3, an autophagy marker related to the autophagosome, was not expressed in the ND group. However, in the HFD group, positive signals were detected in the cytoplasm of lipid-infiltrated hepatocytes (black arrow, Figure 4A). The number of positive signals in the HFD + Evo group was similar to that in the ND group. Immunohistochemical staining for LAMP-1, a lysosome-associated marker of autophagy, also showed a similar expression pattern to LC3 (Figure 4A). To confirm the effects of Evo on autophagy in the liver with HFD, the expressions of Beclin-1 and p62, autophagy markers, were examined. Expression was increased in the HFD group and decreased in the HFD + Evo group (Figure 4B).

### 2.5. Effects of Evogliptin on Hepatic Mitophagy and Oxidative Stress in HFD Mice

**Figure 5:**
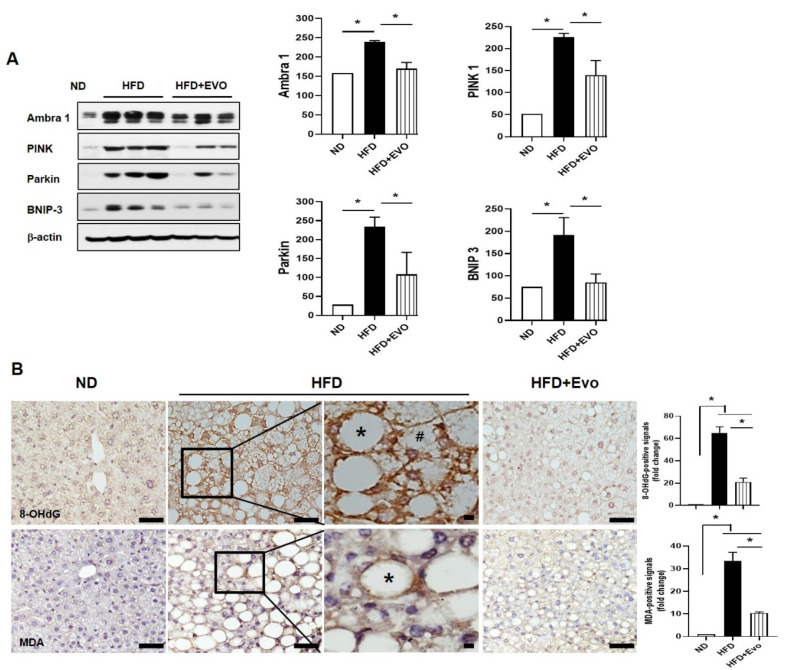
Effect of evogliptin on HFD-induced hepatic mitophagy and oxidative stress: (**A**) the liver tissue lysates from each group were examined by immunoblot with antibodies against mitophagy markers. Each expression is shown as mean ± SEM. * *p* < 0.05. (**B**) Representative immunohistochemical staining for 8-hydroxydeoxyguanosine (8-OHdG) and malondialdehyde (MDA) in liver sections from each group. Scale bar, 100 μm for 8-OHdG and 50 μm for MDA. Oxidative stress, as indicated by 8-OHdG and MDA, was elevated by HFD and decreased by Evo. HFD: high-fat diet; ND: normal diet; # microvesicular steatosis; * macrovesicular steatosis.

It has been reported that mitophagy alleviates hepatic steatosis and that its suppression leads to deterioration of NAFLD [6,7]. The expression of mitophagy markers was significantly increased in the HFD group compared to the ND group. These expressions were significantly lower in the HFD + Evo group compared to the HFD group (Figure 5A). Mitophagy is associated with oxidative stress. Mitophagy is increased in the HFD group which oxyradical-mediated DNA damage (production of 8-hydroxydeoxyguanosine (8-OHdG)) occurs to a greater extent. Immunohistochemical staining showed that the signals of 8-OHdG-positive hepatocytes were increased in hepatocytes with small lipid droplets (#, microvesicular steatosis) as well as large lipid droplets (*, macrovesicular steatosis) of the HFD group compared to the ND group and was decreased in the HFD + Evo group (Figure 5B). To convince that Evo is involved in protection for an oxidative stress by HFD, malondialdehyde (MDA) as a marker of oxidative stress in the sections was also examined. MDA-positive signals are mainly found in hepatocytes with large lipid droplets (*). The signals were increased in the HFD group compared to the ND group and was decreased in the HFD + Evo group (Figure 5B).

### 2.6. Effects of Evogliptin on Hepatic Cell Death by HFD

**Figure 6:**
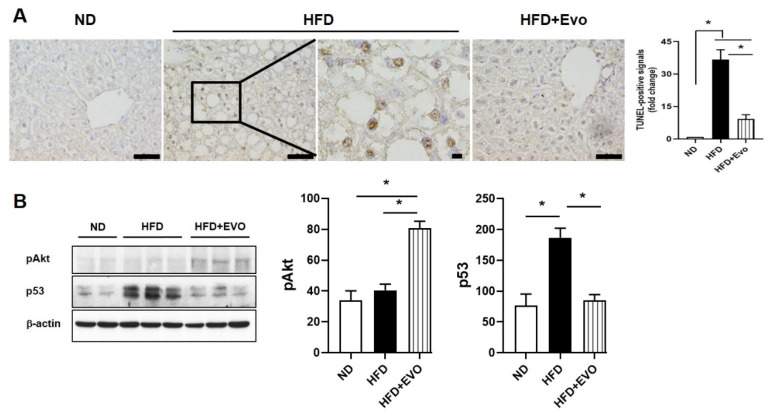
Effect of evogliptin on hepatic cell death by HFD: (**A**) Representative images of TUNEL-stained liver sections from each group. HFD-induced positive signals (black box) were magnified. Scale bar, 50 μm. (**B**) Proteins expressions related to apoptosis and the quantitative results. Values are mean ± SEM. * *p* < 0.05, TUNEL: terminal deoxynucleotidyl transferase dUTP nick end labeling; HFD: high-fat diet; ND: normal diet.

We investigated hepatic apoptosis using a terminal deoxynucleotidyl transferase dUTP nick end labeling (TUNEL) assay (Figure 6A). The number of TUNEL-positive signals was significantly increased in the HFD group compared to the ND group, and most were detected in nuclei of injured hepatocytes. However, in the HFD + Evo group, the number of TUNEL-positive signals was significantly decreased compared to that in the HFD group. Phospho-Akt (pAkt), an antiapoptotic factor, was upregulated and p53, a pro-apoptotic factor, was downregulated in the HFD + Evo group compared to the HFD group (Figure 6B). This data shows that Evo inhibits HFD-induced hepatic cell death by pAkt and p53-involved mechanism.

## 3. Discussion

This study showed that continued lipid stimulus by HFD induced lipotoxicity, fibrosis, autophagy, mitophagy, oxidative stress, and apoptosis in the liver in HFD mice model. However, Evo, a novel DPP-4 inhibitor, attenuated all of these events by HFD.

NAFLD is a chronic inflammatory disorder associated with increased hepatic expression of DPP-4; therefore, inhibition of DPP-4 could ameliorate NAFLD [16]. DPP-4 inhibitors have been reported to decrease lipid accumulation in HFD-induced hepatic steatosis [10,11,12,13,14,17]. However, few studies have examined the mechanism underlying the beneficial effects of DPP-4 inhibitors on NAFLD [17]. Hwang et al. reported that a DPP-4 inhibitor alleviated hepatic steatosis and insulin resistance by increasing AMP-activated protein kinase (AMPK) phosphorylation and by inhibiting leukocyte cell-derived chemotaxin-2 expression [17]. In this study, Evo also reduced lipogenesis by suppressing the Dgat2 mRNA level and improved hyperinsulinemia and liver enzymes. Furthermore, HFD-induced hepatic fibrosis was decreased in the evogliptin treatment group.

Autophagy is generally considered to protect against fatty liver and to degrade damaged cell components in hepatocytes [4]. Exenatide, a glucagon-like peptide 1 analog, delays the progression of NAFLD by promoting autophagy [8]. DPP-4 inhibitors are also shown to induce autophagy, and these lead to beneficial effects such as cardioprotection [18], hepatic insulin resistance, or hepatic steatosis [12]. Zheng et al. also showed that sitagliptin treatment for 4 weeks ameliorated hepatic lipid accumulation via activation of autophagy [19]. We also investigated the effects of Evo on autophagy and mitophagy in HFD-induced steatohepatitis. However, unlike in prior studies [20,21], the expression of autophagy markers was significantly increased in the HFD group and decreased in the HFD + Evo group (Figure 4) in this study. Autophagy is induced in response to various cellular stresses and plays a main role for restoring cellular homeostasis to the pertinent stress [22]. However, beyond a threshold like persistent or excessive stress, such stress causes apoptosis rather than autophagy to eliminate damaged cells [23].

Cellular stress that affects mitochondria can induce a specific autophagy that leads to elimination of damaged mitochondria, namely mitophagy. Mitophagy selectively removes impaired mitochondria and preserves healthy mitochondria [24]. PINK1/Parkin mitophagy is the most studied mitophagy pathway in mammalian cells. Parkin requires PINK1 for translocation to damaged mitochondria to promote mitophagy [24]. Although mitophagy is known to play a protective role in NAFLD [24,25,26], few studies investigated the role of Parkin-mediated mitophagy in liver disease. Williams et al. reported that PINK–Parkin promotes mitophagy and reduces steatosis and apoptosis in an animal model of alcoholic liver disease [25]. However, unlike previous studies, this study shows that expression of mitophagy markers was significantly increased in the HFD group and decreased in the HFD + Evo group (Figure 5). Decreased mitophagy might be associated with improved obesity and steatohepatitis. If improved steatohepatitis is related to decreased mitophagy, the results of Zheng et al. should have shown similar results [19]. However, after sitagliptin treatment of 4 weeks, hepatic lipid accumulation was ameliorated via activation of autophagy in the study of Zheng et al. The results of Shao et al. also showed that exenatide for 4 weeks reduced oxidative stress and hepatic accumulation by enhancing the autophagy/mitophagy pathway [8]. It means that the decreased hepatic steatosis or reduced inflammation is not associated with decreased mitophagy. The discrepancy between our and previous studies [8,19] may be due to the difference in study period. The study duration of ours (16 weeks) was longer than that of Zheng et al. or Shao et al. (4 weeks). Our study showed that TUNEL-positive signals were significantly increased, that the level of pAkt was downregulated, and that p53 was upregulated in the HFD group (Figure 6). Therefore, we thought that persistent HFD stimulus induces stress in the liver; such stress might activate the autophagy pathway with apoptotic cell death rather than cell survival, but less stress is given to the liver by Evo and it finally resulted in reduced hepatic autophagy and mitophagy activation (Figure 6). Autophagy maintains cellular homeostasis but can also promote apoptosis. A study similar to ours was also reported. Liu et al. reported that p53-induced autophagy (primary mitophagy) promotes apoptosis and that mitophagy induces hepatocyte death in vitro [27]. They showed that both oleic acid treatment in vitro and HFD in vivo increased autophagy-induced apoptosis in HepG2 cell and the liver tissue. In addition, autophagy is involved in mitochondrial dysfunction-induced apoptotic death of HepG2 cells [27]. Pang et al. also reported that mitophagy promotes hepatocyte apoptosis [9]. Our data are well correlated with these studies [9,27]. The HFD-induced p53 activation caused autophagy-mediated mitophagy, mitochondrial damage, and cellular oxidative stress and finally leading to apoptosis. However, these effects were attenuated by Evo. To evaluate the precise mechanisms of evogliptin on steatohepatitis and mitophagy, further studies are needed.

In conclusion, Evo alleviates HFD-induced steatohepatitis by inhibiting lipogenesis, fibrosis, and modulating mitophagy in HFD-fed mice. This study suggests that mitophagy could be one of the targets for treating HFD-induced steatohepatitis and that Evo might be used as a therapeutic option in treatment and prevention of NAFLD.

## 4. Materials and Methods

### 4.1. Ethics Statement

This study was approved by the Gyeongsang National University Institutional Animal Care & Ethics Committee (GNU-160804-M0034, 14 May 2018). All animal experiments were performed in accordance with the National Institutes of Health Guide for the Care and Use of Laboratory Animals.

### 4.2. Animals and Treatment

Four-week-old male C57BL/6J mice (Koatech Inc., Peongtaek, Korea) were individually housed with an alternating 12 h light/dark cycle. Mice were divided into three groups and fed for 16 weeks starting at 4 weeks of age with diets as follows: (1) normal diet (ND, 2018S, 3.1 kcal/g, Harlan Laboratories, Inc., Indianapolis, IN, USA) group; (2) high-fat diet (HFD, 45% fat, Research Diets, Inc., New Brunswick, NJ, USA) group; and (3) HFD with evogliptin (HFD + Evo) group. Based on previous studies [28,29], mice were dosed with Evo (250 mg kg^−1^ day^−1^, Dong-A ST Co., Ltd., Seoul, Korea) daily in HFD chow. All mice were weighed weekly and sacrificed at 20 weeks of age. The epididymal fat pad was weighed, and blood and live tissues were harvested. The nutrition table is shown (Table S1).

### 4.3. Tissue Pathology

Mice (*n* = 10 per group) were anesthetized with zoletil (5 mg/kg, Virbac Laboratories, Carros, France). The livers were fixed and processed for paraffin embedding. Next, 5-μm thick sections were cut and stained with H&E for histopathological analysis, and fibrosis was assessed by MT staining. The sections were visualized under a light microscope, and the digital images were analyzed using NIS Elements BR3.2 (Nikon, Tokyo, Japan). Based on a previous study [30], scoring for NAFLD was performed by an experienced pathologist without prior knowledge of the groups using the histological analysis from H&E staining. The score was quantified by summing the scores of lobular inflammation (0–2), macrovesicular and microvesicular steatosis (0–3), and hepatocellular ballooning (0–3). Randomly, 5 fields/section/animal (*n* = 5 per group) were used for the quantification.

### 4.4. Oil Red O Staining and Masson’s Trichrome Staining

To determine hepatic lipid accumulation, frozen liver sections were stained with 0.5% Oil Red O (Sigma) for 10 min, washed, and counterstained with Mayer’s hematoxylin (Sigma) for 45 s. The sections were visualized under a light microscope, and digital images were captured and documented.

### 4.5. Terminal Deoxynucleotidyl Transferase dUTP Nick End-Labeling Assay

Apoptosis was semi-quantitatively assessed by TUNEL assay (Roche, Indianapolis, IN, USA). We counted the numbers of TUNEL-positive cells at 400× magnification in at least 10 random fields per slide and calculated the mean values. Enumeration was performed by a single blinded observer using NIS Elements BR 3.2 (Nikon, Tokyo, Japan) software.

### 4.6. Quantitative Real-Time PCR

The transcript levels of the lipid metabolism-related factors were determined by quantitative real-time (q)PCR. Liver tissues was resuspended in 1 mL of TRIzol Reagent (Invitrogen Life Technologies, Carlsbad, CA, USA), and total RNA was extracted. Purified RNA was subsequently reverse transcribed into cDNA using an iScript cDNA synthesis kit (Bio-Rad Laboratories, Hercules, CA, USA) and oligo-dT primers. After reverse transcription, total DNA was diluted by ddH2O for qPCR. Quantitative cDNA amplification was performed using a ViiA 7 Real-Time System (Applied Biosystems Inc., Foster City, CA, USA). Each of the reaction mixtures contained 9 μL of template cDNA, 10 μL of Taqman Universal Master MixⅡ, no UNG (Applied Biosystems, Foster City, CA, USA), and 1 μL of 20× TaqMan gene expression Assay Mix for the diglyceride acyltransferase 2 (Dgat2, ID:Mm00499536_m1) and patatin-like phospholipase domain containing proteins 2 (Pnpla2, ID:Mm00503040_m1) to a final volume of 20 μL. Thermal cycle conditions were as follows: denaturation at 95 °C for 3 min, followed by 40 cycles of denaturation at 95 °C for 15 s, and annealing and extension at 60 °C for 60 s. GAPDH (ID:Mm99999915_g1) was used as an internal control for the normalization of the quantity of RNA. The relative gene expression level in each sample was quantified using the 2DDCt method.

### 4.7. Protein Preparation and Western Blotting

Tissues were homogenized in lysis buffer, and proteins (50 μg) were loaded and electroblotted. The blots were probed with polyclonal primary antibodies against anti-Ambra1 (ab69501, Abcam, Cambridge, MA, USA), anti-Beclin-1 (sc-48381), p62 (sc-48373), Pink1 (sc-33796), Parkin (sc-30130), BNIP-3 (sc-56167) (Santa Cruz Biotechnology, Santa Cruz, CA, USA), anti-LC3B (#2775), p53 (#9282), and pAkt (#9271) (Cell Signaling Technology, Danvers, MA, USA) at 4 °C overnight. The primary antibody was visualized by adding a secondary antibody and performing an electroluminescence assay (Amersham Pharmacia Biotech, Piscataway, NJ, USA).

### 4.8. Immunohistochemistry

After deparaffinization, sections were incubated with primary antibodies against polyclonal anti-LC3B (#2775, Cell Signaling Technology. Danvers, MA, USA), LAMP-1 (sc-17768, Santa Cruz Biotechnology, Santa Cruz, CA, USA), collagen-III (sc-271249, Santa Cruz Biotechnology, Santa Cruz, CA, USA), 8-OHdG (ab62623, Abcam, Cambridge, MA, USA), and MDA (MDA11-S, Alpha Diagnostic International. San Antonio, TX, USA). Then, biotin-conjugated secondary IgG (1:200 dillution, Vector Laboratories, Burlingame, CA, USA), an avidin–biotin–peroxidase complex (ABC Elite Kit; Vector Laboratories), and DAB were added. Finally, the sections were visualized under a light microscope, and the digital images were analyzed (Elements BR3.2, Nikon, Japan).

### 4.9. Statistical Analysis

Statistical analyses were performed using GraphPad Prism software (v. 8.0; GraphPad Software Inc., La Jolla, CA, USA). Data were evaluated using one-way ANOVA with Tukey’s multiple comparison test (for comparison all groups). A *p*-value < 0.05 was taken to reflect statistical significance. Values are presented as means ± the standard errors of the means.

## Supplementary Materials

Supplementary materials can be found at https://www.mdpi.com/1422-0067/21/18/6743/s1. Table S1. The nutrition table of normal diet and high-fat diet.

## Abbreviations

αSMA	A-smooth muscle actin
ALT	Alanine aminotransferse
AST	Aspartate aminotransferase
Dgat2	Diglyceride acyltransferase 2
DPP-4	Dipeptidyl peptidase-4
HFD	High-fat diet
H&E	Hematoxylin and eosin
MT	Masson trichrome
NAFLD	Nonalcoholic fatty liver disease
ND	Normal chow
8-OHDG	8-hydroxydeoxyguanosine
PINK1	PTEN-induced kinase 1
Pnpla2	Patatin-like phospholipase domain containing proteins 2
pAkt	Phospho-Akt
TG	Triglyceride
TUNEL	Terminal deoxynucleotidyl transferase dUTP nick-end labeling
