# Supplementary material for: Protective Effects of Evogliptin on Steatohepatitis in High-Fat-Fed Mice
Source: Int J Mol Sci. 2020 Sep 14;21(18):6743. doi: 10.3390/ijms21186743 (PMC7555947; doi:10.3390/ijms21186743)
Supplement: Supplementary file 1 [file ijms-21-06743-s001.pdf]

International Journal of
Molecular Sciences

Supplementary Material

Table S1. The nutrition table of normal diet and high fat diet.

Type	Normal Diet	High Fat Diet
Protein	24% Kcal	20% Kcal
Fat	18% Kcal	45% Kcal
Carbohydrate	58% Kcal	35% Kcal
Energy density	3.1 Kcal/g	4.7 Kcal/g
